# Benefit of Sunitinib in the treatment of pulmonary primitive neuroectodermal tumors: a case report and literature review

**DOI:** 10.18632/oncotarget.13896

**Published:** 2016-12-10

**Authors:** Chunhui Zhang, Jingchun Zhang, Guangyu Wang, Jiajia Xu, Yanlin Li, Qing Guo, Tongsen Zheng, Yanqiao Zhang

**Affiliations:** ^1^ Department of Gastrointestinal Medical Oncology, The Affiliated Tumour Hospital of Harbin Medical University, Harbin, Heilongjiang, China; ^2^ Institute of Precision Medicine, 3D Medicines Inc, Shanghai, China

**Keywords:** pulmonary primitive neuroectodermal tumor, next-generation sequencing, von Hippel-Lindau, sunitinib

## Abstract

Primitive neuroectodermal tumor (PNET) is a highly aggressive small round celltumor but is extremely rare in the lung. Next-generation sequencing (NGS) has led to breakthroughs for genetic analyses and personalizedmedicine approaches for cancer treatment.We report the case of a 30-year-old woman with an advanced pulmonary PNET treated with multiple chemotherapeutic regimens, and achieved a partial response (PR) as a best response. However, there was a disease progression after these treatment regimens.The NGS revealed the presence of a copy number loss (CNL) of Von Hippel-Lindau (VHL), CDKN2A/B and TP53 genes. The specific VHL CNL has not previously been associated with PNET, but has been reported in other tumors and has been associated with response to Sunitinib. Sunitinibwas then instituted for this patient and resulted in PR after the failure of multiple chemotherapeutic regimens. To our knowledge, this is the first report of pulmonary PNET with CNL of VHL gene that benefits from Sunitinib treatment. This case illustrates the potential of clinicalNGS to open unexpected avenues for treatment and thereby improve patient outcomes.

## INTRODUCTION

Primitive neuroectodermal tumors (PNET) are rare malignant tumors with poor prognosis, which usually arise from the primitive nerve cells of the nervous system [1]. PNETs occurring outside the central nervous system are commonly referred to as peripheral PNETs [2–3]. PNETs arising from the lung parenchyma without pleural or chest wall involvement are rare [4]. Though the occurrence of pulmonary PNET is rare, these are highly aggressive malignant tumors and metastasize rapidly with poor prognosis due to limited therapeutic options [5]. Importantly, targeted therapies for PNET remain unproven.

**Figure 1:**
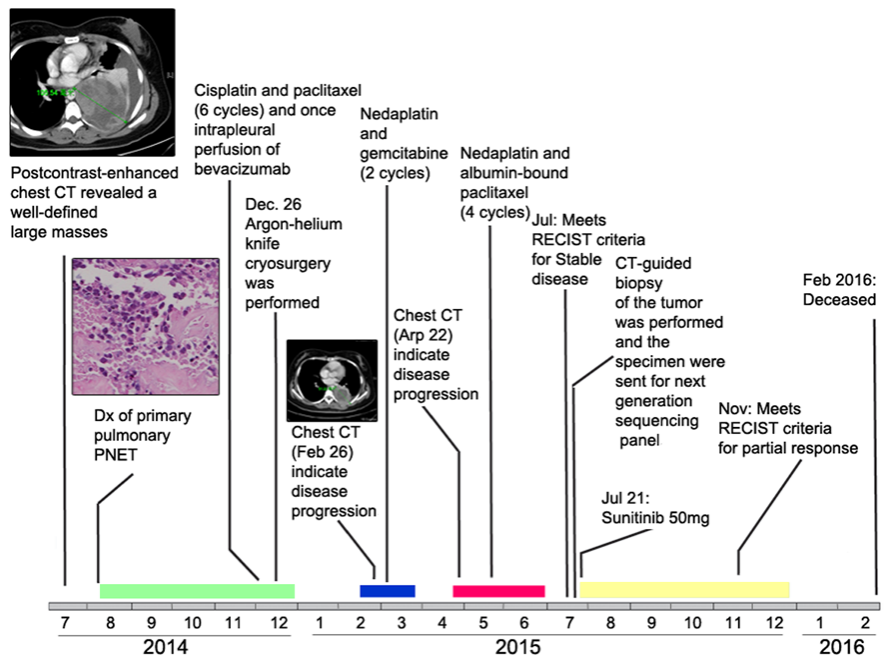
Time line of patient's pulmonary PNET diagnosis, treatment, and response Colored rectangles near the time line represent durations of chemotherapy (green, blue, red) and targeted therapy (yellow). Original magnification, ×200. Dx, diagnosis.

In recent years, there is increasing interest in using individual genetic information to guide cancer treatment, although standard treatment options exist for many cancers [6]. It is possible now to use the genetic changes present in the tumor detected by next-generation sequencing (NGS) to guide the selection of target therapies [4]. It is especially useful in treatment-refractory patients for which standard therapeutic options have failed. We herein present a case of pulmonary PNET in which NGS exhibited a therapeutic target of Von Hippel-Lindau (VHL) copy number loss (CNL) that failed multiple chemotherapy regimens and argon-helium knife cryosurgery. Therapy directed against the target gene CNL resulted in a partial response (PR) of previously progressive disease (Figure 1). To the best of our knowledge, this is the first case report of primary pulmonary PNET with VHL gene CNL that benefits from Sunitinib treatment.

## CASE REPORT

### Presenting concerns

A thirty years old woman was admitted to the Affiliated Tumor Hospital of Harbin Medical University in July 2014 with the complaints of chest tightness, shortness of breath and chest pain of ten-day duration. She denied smoking and had no family history of malignancies. She had no other complaints and her past history was unremarkable. Tumor biomarker tests (alpha fetoprotein, antigen 19-9, carcinoembryonic antigen, prostate-specific antigen, Cytokeratin 19 Fragment, carbohydrate antigen 24-2, squamous cell carcinoma antigen, and neuron-specific enolase) were all normal on admission, as were the baseline serum chemistry screening, peripheral blood count and the urinalysis.

### Diagnostic focus and assessment

**Figure 2:**
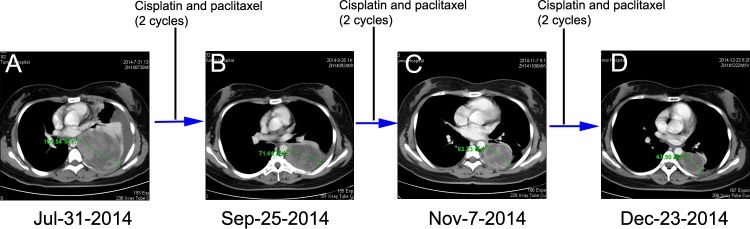
Chest CT scans documenting responses to cisplatin and paclitaxel (CP) chemotherapy **A.** Chest CT at the initial presentation reveals showing a pace-occupying mass in the left lung (July 31, 2014). Chest CT scan was taken after every two cycles of CP **B.** and **C.** After six cycles of CP treatment, CT image showing a PR (Dec 23, 2014) by RECIST **D.**.

**Figure 3:**
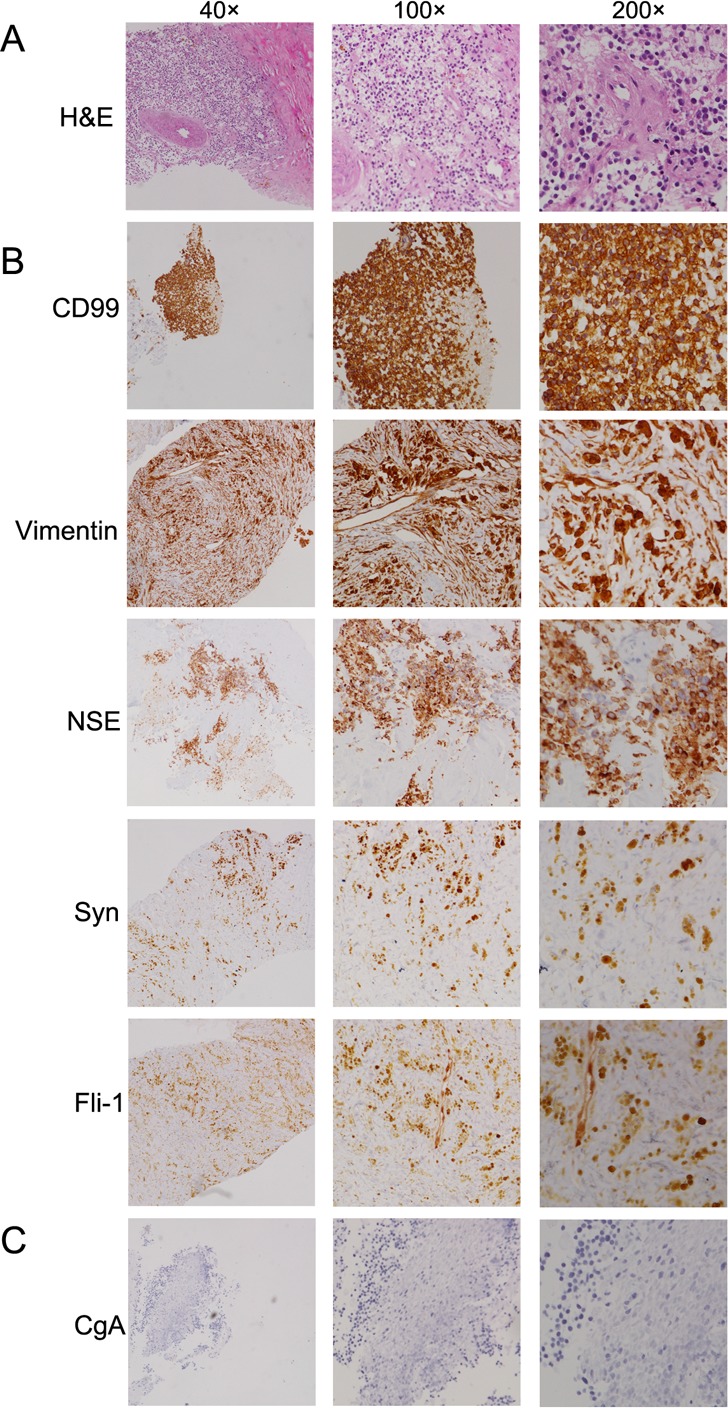
Histologic features of the pulmonary PNET **A.** Hematoxylin-eosin (H&E) staining of the primary pulmonary primitive neuroectodermal tumor. **B.** and **C.** Immunohistochemistry staining of the primary tumor with different antibodies, such as anti-CD99, vimentin, NSE, synaptophysin (Syn), friend leukemia integration 1 transcription factor (Fli-1) and chromogranin A (CgA).

Chest computed tomography (CT) scan (July-31, 2014) revealed a heterogenous mass that measured 105.54 mm in longest diameter in the left lower lobe lung with pleural effusion and left lower lobe collapse (Figure 2A). Enhanced CT showed the mass obviously enhanced with heterogeneous density and obscure boundary. There were some areas of necrosis within the mass and it was extending up to the pleural surface. To establish the pathological diagnosis, a CT-guided biopsy of the mass was performed and sent for pathological evaluation. The histopathologic results showed small, round tumor cells that exhibited mild morphology; arranged in cords and embedded in fibrous tissue (Figure 3A). The immunostaining showed positive expression of vimentin, CD99, synaptophysin (Syn), friend leukemia integration 1 transcription factor (Fli-1) and NSE (Figure 3B), and negative expression of Chromogranin A (CgA) (Figure 3C), cytokeratin 7 (CK7), protein S-100 (S-100), thyroid transcription factor 1 (TTF-1), CD56, CD20, CD3, CD10, and p63 (Figure S1).

### Therapeutic focus and assessment

**Figure 4:**
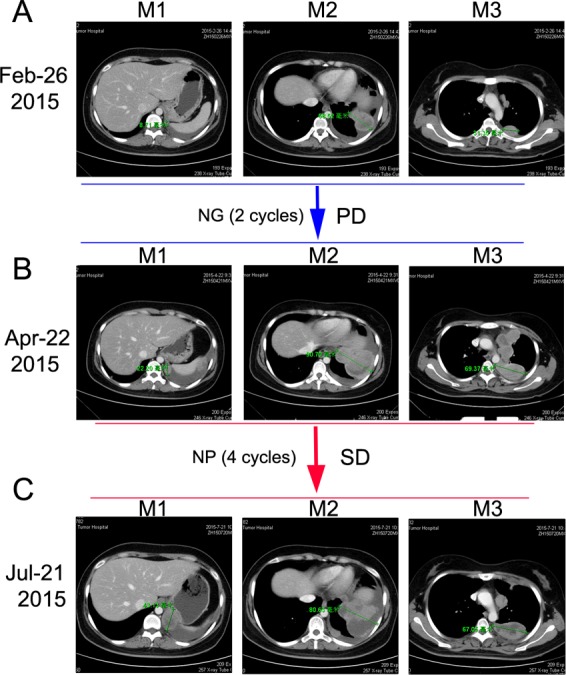
Chest CT scans documenting responses to nedaplatin and gemcitabine (NG) and nedaplatin and albumin-bound paclitaxel (NP) chemotherapies **A.** Baseline CT image before NG treatment. **B.** After two cycles of NG treatment, CT image showing a PD. **C.** After 4 cycles of NP treatment, CT image showing a SD.

The patient was initially treated with traditional chemotherapeutic regimen that consisted of cisplatin/paclitaxel (CP, cisplatin 270mg/m^2^ and paclitaxel 60mg/m^2^) and once intrapleural perfusion of bevacizumab (200mg) and then five additional cycles of CP alone. Chest CT scan was taken after every 2 cycles of CP treatment, which showed the regimen was effective (Figure 2B and 2C). After 6 cycles of CP treatment, the mass measured 47.90 mm on CT (Dec 23, 2014), which indicated a 54.61% decrease compared with baseline, and met the RECIST (Response Evaluation Criteria in Solid Tumors) criteria for a partial response (PR) (Figure 2D). Argon-helium knife cryosurgery was then performed (Dec 26, 2014) however, multiple metastases were detected two months after the surgery (Jan 29, 2015). Chest CT scan (Feb 26, 2015) indicated clear evidence of disease progression, with left pleural metastases, left pleural effusion, mediastinal lymphadenopathy, and invasion of back muscles and ribs. As a cryosurgery has been performed on the main lesion, we then choose three main pleural metastases as targeted lesions (Figure 4A). Since progressive disease was apparent, the chemotherapy regimen was changed to nedaplatin/gemcitabine (NG, nedaplatin 60mg/m^2^ and gemcitabine). After 2 cycles of NG treatment, chest CT scan (Apr 22, 2015) revealed that the disease continued to progress (Figure 4B). The regimen was then changed to the third line nedaplatin/albumin-bound paclitaxel (NP, nedaplatin 60mg/m^2^ and albumin-bound paclitaxel 200mg/m2) therapy subsequently. Chest CT scan (July 21, 2015) after 4 cycles of NP treatment suggested a stable disease (SD) (Figure 4C). However, the regimen was discontinued because of severe neurotoxicity.

**Figure 5:**
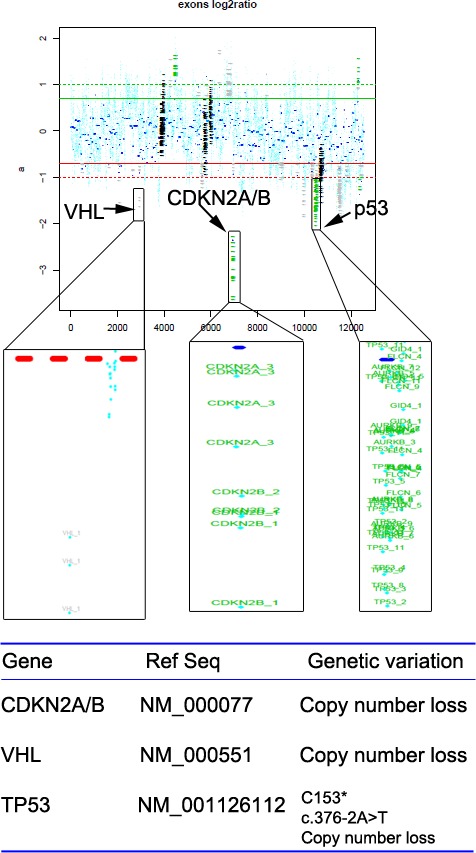
Copy number loss of VHL, CDKN2A/B and TP53 genes were detected in the tumor tissues of the pulmonary PNET patient

**Figure 6:**
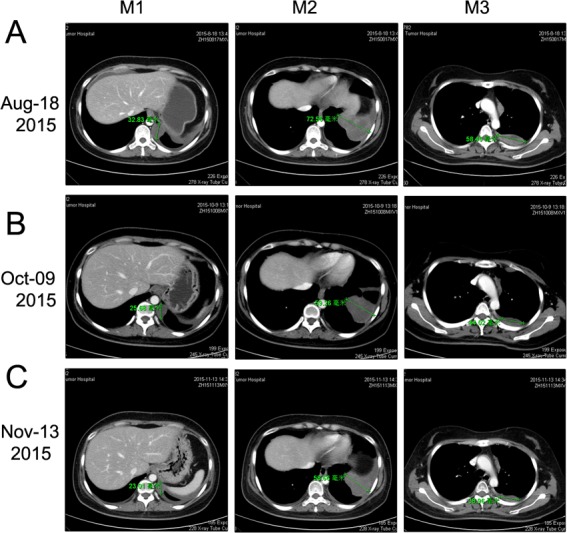
Chest CT scan documenting responses to Sunitinib **A.** After 4 weeks of treatment with Sunitinib (50 mg/day), CT image showing a SD (-14.12%, Aug 18, 2015). **B.** After about three months of treatment with Sunitinib, CT image showing a PR (-32.41%, Oct 09, 2015). **C.** After continuous treatment with Sunitinib for about four months, CT image showing a confirmed PR (-36.78%, Nov 13, 2015).

To explore the genomic profiling of the target lesion for target therapy, CT-guided biopsy of the tumor was performed, the tissue specimen and matched blood sample were sent for NGS panel after consent obtained from the patient and his family (May 22, 2015). We detected all genomic alteration types, including deletions and insertions, base substitutions and copy number alterations on over 390genes commonly associated with cancers (Table S1). The genomic profile of the tumor revealed CNL of VHL, CDKN2A/B and TP53 genes (Figure 5). Since there was a CNL of VHL gene in this patient, it was reasonable to use bevacizumab, sorafenib or Sunitinib for target therapy. Given the disease progressed after multiple lines of chemotherapies potentially actionable VHL CNL, the patient was started on Sunitinib treatment (Jul-21, 2015). CT scan (Aug 18, 2015) indicated a SD by RECIST with a 14.12% decrease in the sum of longest diameters of the target lesions compared with the baseline of July 21 (Figure 3C →Figure 6A), and the chest pain relieved significantly. After three months of Sunitinib therapy, CT scan showed a PR (Oct 9, 2015) by RECIST, with a 32.4% decrease in the sum of longest diameters of the target lesions (Figure 3C →Figure 6B). And the Sunitinib therapy achieved a confirmed PR on Nov 13, 2015 (M1 lesion 43.13→23.01mm; M2 lesion 80.63→58.66mm; M3 lesion 67.05→38.96mm; Figure 3C→Figure 6C). However, the regimen was discontinued because of severe toxicity, including bone marrow suppression (Grade IV) and hemorrhage. The patient's mental status and physical status worsened day by day. About three months after the stop of the Sunitinib treatment, the patient died.

Furthermore, we have reviewed the literatures of primary pulmonary PNETs in Table S2 [7–19]. There were less than 30 PNET cases reported in the literatures. Presenting symptoms included cough, chest pain, dyspnea, and hemoptysis [4]. The diagnosis was usually made based on the typical morphological appearance characterized by a homogeneous population of closely packed small blue round cells and occasional Homer-Wright-type rosettes [8]. Histochemical and immunohistochemical studies were performed to confirm the diagnosis by demonstrating positivity of tumor cells with CD99, vimentin, NSE, and occasional periodic acid Schiff reactivity [10–16]. There were 13 cases with the overall survival in these 24 cases, ranging from 1 to 54 months, while 9 patients were still alive with a follow up ranging from 9 to 34 months. Seventeen cases underwent resection plus adjuvant chemotherapy or neoadjuvant chemotherapy.

## DISCUSSION

PNET is an group of tumors comprised of small round cells, and remote metastases and local recurrence are common [20]. PNET is usually diagnosed by histologic and immunohistochemical analysis. It was histologically composed of small round cells with scanty cytoplasm [13–16]. The chromosomal translocation t (11;22) (q24;q12) is an additional firm diagnostic criterion. For the patient, as the staining of NSE, CD99, and vimentin was positive and the diagnosis of PNET was confirmed. As reported, multimodality treatment, including surgical resection, radiotherapy, neoadjuvant and adjuvant chemotherapy, is the treatment of choice for PNETs [21]. Moreover, relevant studies are mostly single-institution, small-scale clinical studies, due to the rarity of this disease. The diagnosis and treatment of pulmonary PNET still remains a challenge for the clinic due to the absence of guidelines.

The patient reported in this study presented with an advanced pulmonary PNET underwent multiple therapeutic regimens including chemotherapy and argon-helium knife cryosurgery, and achieved PR as a best response. However, disease progressed after these treatment regimens, and the patient commenced targeted therapy. As multiple chemotherapy regimens have been performed, there might be some treatment related mutations, which should not be discounted. It should also be interesting to perform the genomic analysis on index sample for earlier targeted therapy in the future. For the present case, we intended to identify the possible targetable molecular alterations before we can start the targeted therapy, therefore we made the decision to send tumor tissue from the lung after disease progression for NGS of a gene panel in which genetic variation is known to be associated with the response to therapies. Fortunately, we got enough tumor tissues for the NGS test, which might be due to the progression of the disease. The NGS results revealed somatic CNL of VHL, CDKN2A/B and TP53 genes.

So far, there has been no FDA approved drugs targeting VHL, but agents targeting the vascular endothelial growth factor (VEGF) pathway have been approved to have value in the treatment of VHL-related tumors, including bevacizumab and multi-kinase inhibitors, such as Sunitinib and sorafenib [22]. Sunitinib is an oral multitargeted receptor tyrosine kinase inhibitor that targets platelet-derived growth factor receptor (PDGFR), FLT3, KIT, and VEGFR [23]. In malignancies such as kidney cancer and pancreatic neuroendocrine tumors where a therapeutic role of Sunitinib has been established [23–25], the absence of the VHL gene causes accumulation of HIFs and the production of several growth factors, including VEGF and PDGF against which Sunitinib has efficacy [23]. So it was inferred that the patient with CNL of VHL may benefit from Sunitinib. Additionally, there is a recent report suggested that one PNET patient treated with Sunitinib showed a significant radiological response with almost complete necrosis of the perihepatic metastatic lesion and a progression-free survival of nine months [23]. In another study, a case of PNET arising in the kidney during pregnancy treated with sorafenib was fairly well tolerated, a thoracic CT scan revealed a 40%-volumetric regression of lung metastasis [26]. These reports indicated that Sunitinib or sorafenib might be both effective in the treatment of pPNET. Importantly, there are lots of reports suggested that Sunitinib exhibited excellent efficiency in different cancers with VHL disease, which results from a mutation in VHL gene [27, 28]. Therefore, the reason we choose Sunitinib over sorafenib is that we found this PNET patient here has a CNL of VHL, which suggested that the PNET patient might get more treatment benefits from Sunitinib over sorafenib. As for bevacizumab, although there is one study reported that bevacizumab was effective in advanced uterine PNET [29], however, there is some report suggested that bevacizumab showed no efficacy on PNET [30]. Therefore, the efficiency of bevacizumab on PNET is still controversial. Actually, we have already used bevacizumab once for this patient, but the patient refused to receive bevacizumab treatment because of adverse effects (hypertension and diarrhea). Additionally, bevacizumab is a recombinant humanized monoclonal antibody that blocks angiogenesis by inhibiting only VEGF-A [29, 30], whereas sorafenib and Sunitinib are inhibitors of multiple kinases, such as VEGFR and PDGFRs [24]. In addition, a recent study reported that there was a MLH1 gene mutation in a PNET patient, they therefore inferred that vastin or Cetuximab could be used for the target therapy [4]. However, the patient did not have the opportunity to receive this target therapy. In our case, four-month treatment of Sunitinib achieved PR according to RECIST criteria (Figure 1) and improved the quality of life (QoL) significantly. These results are very interesting, considering that the patient was heavily pretreated before the targeted therapy.

Besides VHL, targeted NGS revealed CNL of CDKN2A/B and TP53 genes in this patient as well. CDKN2A gene produces two unrelated tumor suppressors, p16INK4a and p14ARF4, and CDKN2B gene produces tumor suppressor p15INK4b [31–32]. No drugs targeting CDKN2A/B have been approved, while CDK4/6 inhibitor paboxilin has been proved in breast cancer by FDA. Various experiments revealed that paboxilin inhibited cell growth or mice tumor proliferation with CDKN2A deletion selectively and sensitively, which indicated that CDKN2A/B CNL may respond to CDK4/6 inhibitor [33]. TP53 gene produces tumor suppressor p53 that is either lost or mutated in various malignancies [34]. Also, no drugs targeting TP53 have been approved by FDA, while it was indicated that TP53 mutation was sensitive to Wee-1 inhibitor MK-1775 [34]. Therefore, paboxilin, MK-1775 or other targeted drugs may serve as an alternative if the patient failed to response to Sunitinib treatment.

In conclusion, the outcome of pulmonary PNET is extremely poor partly due to the limitation of traditional therapies. The current study is the first case report of pulmonary PNET that benefits from Sunitinib treatment. Moreover, it demonstrates the feasibility of target therapy in the treatment of this rare cancer.

## SUPPLEMENTAL INFORMATION FIGURE AND TABLE


