# Supplementary material for: Benefit of Sunitinib in the treatment of pulmonary primitive neuroectodermal tumors: a case report and literature review
Source: Oncotarget. 2016 Dec 10;7(52):87543–51. doi: 10.18632/oncotarget.13896 (PMC5350009; doi:10.18632/oncotarget.13896)
Supplement: Supplementary file 1 [file oncotarget-07-87543-s001.pdf]

# Benefit of Sunitinib in the treatment of pulmonary primitive neuroectodermal tumors: a case report and literature review

## Supplemental Information

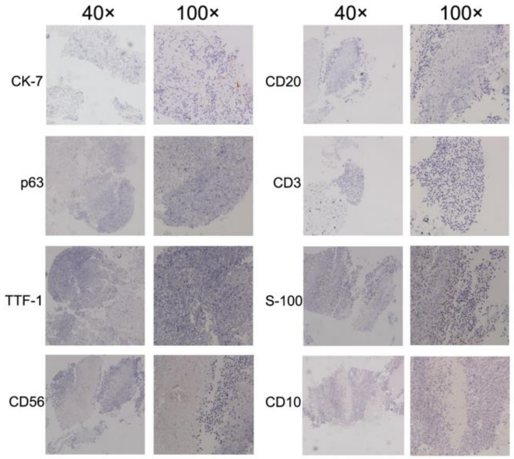


**Figure S1:**

Histologic features of the primary pulmonary PNET. The images for the immunohistochemistry staining of cytokeratin 7 (CK7), p63, thyroid transcription factor 1 (TTF-1), CD56, CD20, CD3, CD10 and protein S-100 (S-100).

**Table S1:**

All genomic alteration types, including base substitutions, insertions and deletions, copy number alterations and rearrangements on a panel of 390 genes commonly associated with cancers were detected for the pulmonary PNET patient.

| **Gene mutation** | | | | | | | | | | | | | |
|---|---|---|---|---|---|---|---|---|---|---|---|---|---|
| **ABL1** | BCL2L2 | CDK4 | DDR2 | FAS | GATA4 | INPP4B | MAGI2 | NF2 | PIK3CG | RBM10 | SOX9 | TSC2 |
| **ABL2** | BCL6 | CDK6 | DICER1 | FAT1 | GATA6 | IRF2 | MAP2K1 | NFE2L2 | PIK3R1 | RET | SPEN | TSHR |
| **ACVR1B** | BCOR | CDK8 | DNMT3A | FBXW7 | GID4 | IRF4 | MAP2K2 | NFKBLA | PIK3R2 | RICTOR | SPOP | TYK2 |
| **ACVR2A** | BCORL1 | CDKN1A | DOTIL | FGF10 | GLI1 | IRS2 | MAP2K4 | NKX2-1 | PKD2 | RNF43 | SPTA1 | U2AF1 |
| **ADAM29** | BIRC5 | CDKN1B | EGF | FGF14 | GLI2 | ITK | MAP3K1 | NOTCH1 | PLA2G1B | ROCK1 | SRC | VEGFA |
| **AKT1** | BLK | CDKN2A | EGFR | FGF19 | GLI3 | JAK1 | MAP4K5 | NOTCH2 | PLCG2 | ROCK2 | SRMS | VHL |
| **AKT2** | BLM | CDKN2B | EP300 | FGF23 | GNA11 | JAK2 | MCL1 | NOTCH3 | PMS2 | ROS1 | STAG2 | WEE1 |
| **AKT3** | BMX | CDKN2C | EPHA2 | FGF3 | GNA13 | JAK3 | MDM2 | NPM1 | POLD1 | RPTOR | STAT3 | WEE2 |
| **ALK** | BRAF | CEBPA | EPHA3 | FGF4 | GNAQ | JUN | MDM4 | NRAS | POLE | RUNX1 | STAT4 | WISP3 |
| **AMER1** | BRCA1 | CHD2 | EPHA5 | FGF6 | GNAS | KAT6A | MED12 | NRG1 | PPP2R1A | RUNX1T1 | STK11 | WT1 |
| **APC** | BRCA2 | CHD4 | EPHA7 | FGFR1 | GPR124 | KDM5A | MEF2B | NRG3 | PRDM1 | RXRA | STK24 | XIAP |
| **AR** | BRD4 | CHEK1 | EPHB1 | FGFR2 | GRIN2A | KDM5C | MEN1 | NSD1 | PREX2 | SDHA | SUFU | XPO1 |
| **ARAF** | BRIP1 | CHEK2 | ERBB2 | FGFR3 | GRM3 | KDM6A | MET | NTRK1 | PRKAR1A | SDHB | SYK | YES1 |
| **ARFRP1** | BTG1 | CIC | ERBB3 | FGFR4 | GSK3B | KDR | MITF | NTRK2 | PRKCI | SDHC | TAF1 | ZBTB2 |
| **ARID1A** | BTK | CRBN | ERBB4 | FGR | H3F3A | KEAP1 | MLH1 | NTRK3 | PRKDC | SDHD | TBX3 | ZNF217 |
| **ARID1B** | C11orf30 | CREBBP | ERCC1 | FH | HCK | KEL | MPL | NUP93 | PRSS8 | SETD2 | TCF7L2 | ZNF703 |
| **ARID2** | CARD11 | CRKL | ERG | FLCN | HGF | KIT | MRE11A | PAK3 | PTCH1 | SF3B1 | TEK | ZNF750 |
| **ASXL1** | CBFB | CRLF2 | ERRFI1 | FLT1 | HNF1A | KLHL6 | MS4A1 | PALB2 | PTEN | SIK1 | TET2 | |

| | | | | | | | | | | | | |
|---|---|---|---|---|---|---|---|---|---|---|---|---|
| **ATM** | CBL | CSF1R | ESR1 | FLT3 | HRAS | KMT2A | MSH2 | PARK2 | PTK2 | SLIT2 | TGFBR1 | |
| **ATR** | CCND1 | CSK | EZH2 | FLT4 | HSD3B1 | KMT2C | MSH6 | PAX5 | PTK6 | SMAD2 | TGFBR2 | |
| **ATRX** | CCND2 | CSNK1A1 | FAM135B | FOXL2 | HSP90AA1 | KMT2D | MST1R | PBRM1 | PTPN11 | SMAD3 | TIE1 | |
| **AURKA** | CCND3 | CTCF | FAM46C | FOXP1 | IDH1 | KRAS | MTOR | PDCD1LG2 | QKI | SMAD4 | TNFAIP3 | |
| **AURKB** | CCNE1 | CTNNA1 | FANCA | FRS2 | IDH2 | LCK | MUTYH | PDGFRA | RAC1 | SMARCA4 | TNFRSF14 | |
| **AXIN1** | CD274 | CTNNB1 | FANCC | FUBP1 | IGF1R | LIMK1 | MYC | PDGFRB | RAD50 | SMARCB1 | TNFSF11 | |
| **AXL** | CD79A | CUL3 | FANCD2 | FYN | IGF2 | LMO1 | MYCL | PDK1 | RAD51 | SMO | TNK2 | |
| **BAP1** | CD79B | CXCR4 | FANCE | GABRA6 | IKBKE | LRP1 | MYCN | PIK3C2B | RAF1 | SNCAIP | TOP1 | |
| **BARD1** | CDC73 | CYLD | FANCF | GATA1 | IKZF1 | LRP1B | MYD88 | PIK3CA | RANBP2 | SOCS1 | TOP2A | |
| **BCL2** | CDH1 | DAXX | FANCG | GATA2 | IL7R | LYN | NEK11 | PIK3CB | RARA | SOX10 | TP53 | |
| **BCL2L1** | CDK12 | DDR1 | FANCL | GATA3 | INHBA | LZTR1 | NF1 | PIK3CD | RB1 | SOX2 | TSC1 | |

| **Fusion Gene** | | | | | | | | | | | | |
|---|---|---|---|---|---|---|---|---|---|---|---|---|
| **ALK** | BCR | BRAF | BRCA1 | BRCA2 | BRD4 | ETV1 | ETV4 | ETV5 | ETV6 | FGFR1 | FGFR2 | FGFR3 |
| **MYB** | NOTCH2 | NTRK1 | NTRK2 | PDGFRA | RAF1 | RARA | RET | ROS1 | TMPRSS 2 | MET | DDR2 | |

**Table S2. Primary Pulmonary Primitive Neuroectodermal Tumor Patients Reported in the Literatures**

| Year | Age | Gender | Treatment | Follow-up | Reference |
|---|---|---|---|---|---|
| 2015 | 26 | M | R/ad CRT | DOD at 3M | 7 |
| 2015 | 16 | M | R/ad CRT | DOD at 5M | 4 |
| 2014 | 31 | F | CT | DOD at 1M | 8 |
| 2013 | 75 | M | CT | A&W at 17M | 9 |
| 2013 | 31 | | R/ad CRT | DOD at 36M | 10 |
| 2012 | 22 | M | R only | NK | 11 |
| | 27 | M | R/ad CRT | DOD at 24M | |
| | 29 | F | R/ad CT | DOD at 36M | |
| | 31 | M | R/ad CT | DOD at 54M | |
| | 29 | M | R only | NK | |
| | 56 | F | Neoad CT/R/ad CT | A&W at 11M | |
| 2010 | 44 | M | R/ad CT | DOD at 5M | 12 |
| 2009 | 22 | F | Neoad CT/R/ad CRT | A&W at 32M | 13 |
| | 28 | F | R/ad CT | A&W at 18M | |
| | 22 | M | R/ad CT | DOD at 18M | |
| | 47 | M | Neoad CT/R/ad CT | A&W at 34M | |
| 2007 | 8 | M | R/ad CT | A&W at 9M | 14 |
| 2001 | 17 | M | R/ad CRT | DOD at 9M | 15 |
| 2001 | 18 | M | R only | DOD at 24M | 16 |

| | | | | | |
|---|---|---|---|---|---|
| 2001 | 26 | F | Neoad CT/R/ad CRT | DOD at 18M | 17 |
| 2000 | 30 | F | Neoad CT/R/ad CT | A&W at 16M | 18 |
| | 41 | M | Neoad CT/R | A&W at 22M | 19 |
| 1998 | 25 | F | R only | DOD at 24M | |
| | 15 | M | R only | A&W at 24M | |
